# Harmonisation of Outcome Parameters and Evaluation (HOPE) for actinic keratosis: protocol for the development of a core outcome set

**DOI:** 10.1186/s13063-019-3696-6

**Published:** 2019-10-11

**Authors:** Markus V. Heppt, Theresa Steeb, Lutz Schmitz, Claus Garbe, Lars E. French, Ulrike Leiter, Carola Berking

**Affiliations:** 1 Department of Dermatology and Allergy, University Hospital, LMU Munich, Frauenlobstr. 9–11, 80337 Munich, Germany; 2 Department of Dermatology, Venereology and Allergology, Ruhr-University, Gudrunstr. 56, 44791 Bochum, Germany; 3 Department of Dermatology, Center for Dermatooncology, University Hospital Tübingen, Liebermeisterstr. 25, 72076 Tübingen, Germany

**Keywords:** Actinic keratosis, Core outcome sets, Delphi, Focus groups, Randomised controlled trials

## Abstract

**Background:**

Actinic keratoses (AK) are common skin lesions that can progress to invasive squamous cell carcinoma of the skin. A variety of lesion- or field-targeted treatment options exist and their efficacy has been demonstrated in numerous randomised controlled trials (RCTs). However, the reported endpoints are highly heterogeneous, making it difficult to assess and compare distinct treatment options and to reach an evidence-based choice of therapy.

**Methods:**

A systematic literature search will be conducted to analyse which endpoints are reported in RCTs. The focus will be on effectiveness, tolerability, cosmesis, and patient satisfaction. The reported endpoints of these studies, as well as their frequency and data collection times, will be documented in a standardised way to generate a comprehensive list of reported endpoints. In order to complete the identified outcomes in the literature search, focus groups on affected patients and structured interviews with board-certified dermatologists will be conducted to identify both patient- and practice-relevant endpoints. After the identification phase, the evaluation of the endpoints follows. In a two-stage Delphi procedure, experts including patient representatives will evaluate the endpoints in a standardised and transparent manner. A final face-to-face consensus meeting will be conducted after the last Delphi round in which a final list of core outcomes will be consented.

**Discussion:**

The development of a standardised endpoint set for the treatment of AK will contribute to improving the comparability of therapeutic options. Our catalogue will enhance the synthesis of evidence for the future by reducing heterogeneity in outcomes between RCTs and hence contribute to improving the quality of research, evidence-based and patient-centred treatment.

**Trial registration:**

Core Outcome Measures for Effectiveness (COMET) database. Registered in December 2018.

## Background

Actinic keratoses (AKs) are precancerous lesions of the skin as a consequence of long-term sun exposure [1, 2]. They are among the most common skin lesions with a prevalence of up to 50% in white-skinned people over the age of 70 years. AKs can progress into cutaneous squamous cell carcinoma (cSCC), although the risk is presumably low [3]. International guidelines recommend the treatment of AKs since it is not possible to predict whether a lesion will become an invasive cSCC [4]. A variety of lesion- or field-targeted treatment options exist and their efficacy has been demonstrated in numerous randomised controlled trials (RCTs). However, the reported endpoints are highly heterogeneous in terms of efficacy, tolerability, cosmetic outcome, and patient satisfaction, making it difficult to assess and compare treatment options.

Therefore, we are aiming at developing a standardised core catalogue of the most relevant and suitable endpoints for treatment evaluation in AK lesions through a multi-step project. This core outcome set may contribute to improve the comparability of distinct treatments and aid physicians to make an appropriate treatment choice in daily practice.

### Objective

The primary objective of this endeavour is to identify and consent a catalogue of endpoints of AK treatment and to coin their associated definitions for a standardised reporting and evaluation of AK therapy in clinical practice.

## Methods

**Fig. 1:**
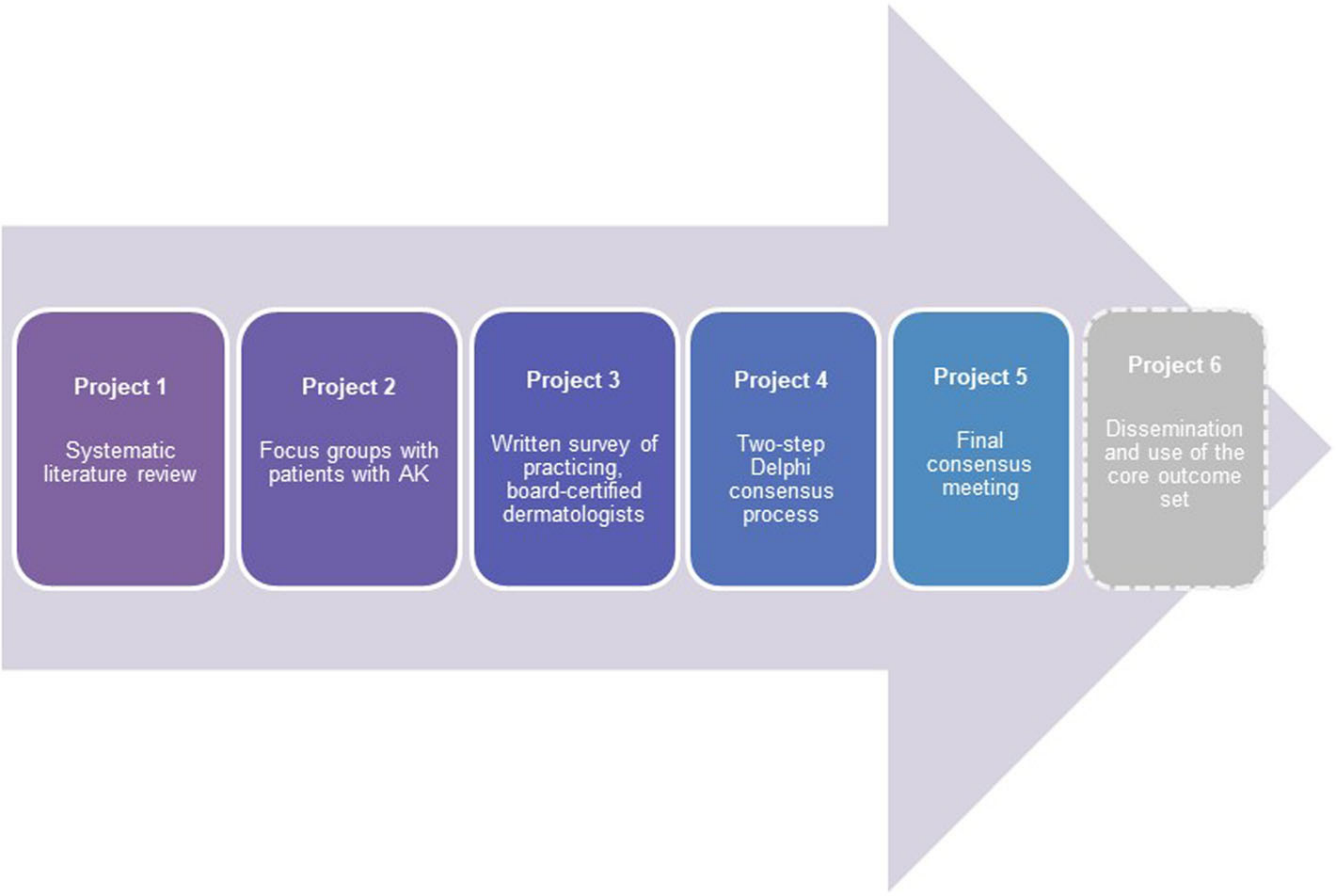
Flowchart and sub-projects of the HOPE initiative

The design of this project will be guided by the Core Outcome Measures for Effectiveness (COMET) Handbook [5]. A step-wise refinement approach will be used, consisting of six discrete, yet complementary, sub-projects (Fig. 1).

- Project 1: A comprehensive literature review to identify reported outcomes in RCTs evaluating interventions for AKs
- Project 2: Focus groups with patients with AKs
- Project 3: Written survey of practicing, board-certified dermatologists with a piloted questionnaire
- Project 4: Two-step Delphi consensus process
- Project 5: In-person meeting to reach a final consensus on a core outcome set
- Project 6: Publication and dissemination of the catalogue of core endpoints

### Step 1: Comprehensive literature review to identify reported outcomes in RCTs evaluating interventions for AK

#### Objective

To analyse which endpoints are reported in published RCTs. The focus will be on effectiveness, tolerability, cosmesis, and patient satisfaction. The conduct of the review will adhere to standard searching and selection strategies.

#### Inclusion criteria

Patients with the clinical and/or histopathological diagnosis of AKs will be included. We will only include RCTs in which study participants (inter-individual trials) or entire body parts (intra-individual trials) are investigated. Pseudo-randomised trials, observational studies, retrospective studies, cross-over studies, and case series will be excluded.

#### Search methods for identification of studies

We will search the electronic databases Medline, Embase (both via Ovid), and the Cochrane library CENTRAL from their inception to identify all relevant records. The combinations of keywords and terms that will be used in the search are major Medical Subject Headings (MeSH) terms and AK-specific keywords. No language restrictions will be applied. The search queries are available in Additional file 1.

#### Data collection and management

Two investigators (TS, MVH) will independently screen titles and abstracts for eligibility that have been identified in the electronic database searches. For records that are considered relevant according to title and abstract screening, full-text articles will be obtained, and inclusion and exclusion criteria will be applied. Whenever discrepancies arise, resolution will be achieved by discussion with a third independent investigator (LS, CG, UL, LEF, CB). A purposively developed data abstraction form in Microsoft Excel 2010 will be used to collect the baseline characteristics of the eligible studies, including author, year of publication, study design, number of arms, interventions, sample size, and duration of follow-up. We will collect all outcomes in the included RCTs that have been previously identified in the Cochrane review by Gupta et al. [6]. Reported endpoints will be categorised as efficacy, safety, cosmesis or patient satisfaction outcomes. Besides, we will also collect the time of assessment of the outcomes. If incomplete reporting is noticed in the primary studies, the outcomes that are described in the ‘Methods’ section of the relevant study will be included as well as the outcomes for which findings are presented.

#### Data analysis

Once data from all included studies have been extracted, a list of published AK-specific outcomes and their definitions will be developed. Descriptive analysis will be performed with expression of frequencies, means, and median.

### Step 2: Focus groups on patients affected by AK to identify patient-relevant outcomes

#### Objective

To explore AK-specific endpoints which are relevant for patients with AKs in order to complete the list from step 1.

#### Design

In order to complete the identified outcomes from the literature search, a qualitative and explorative design will be used to identify patient-related and, particularly, practice-relevant endpoints through focus group discussions. Qualitative approaches enable an in-depth picture of patients’ preferences and needs [7, 8]. Besides, the interactive component of the focus groups enables participants to ponder, reflect and listen to the experiences and opinions of others [9]. The interview will be structured according to published guidelines for focus groups [9]. The manual with the questions for the conduction of the focus groups will be developed by the investigators of this study and will be based on dermato-oncologic experience, a Cochrane review [6], and a guideline for interventions for AKs [4]. The survey and the interviews in the focus groups will contain items on different therapy dimensions (effectiveness, tolerability, cosmesis, patient satisfaction) and should determine which endpoints are perceived as particularly important by patients. Follow-up and probing questions will be used for clarification and elaboration. Besides, demographic data, such as age or sex, will be obtained from the participants.

#### Sampling, recruitment, and data collection

Participants will be recruited via German patient support groups (e.g. https://hautkrebs-netzwerk.de/), local Facebook™ groups as well as flyers in the hospital and direct contact by attending physicians in the Oncological Outpatient Department of the University Hospital (LMU Munich, Germany). Patients who have already been diagnosed with AKs and who have undergone at least one therapy in their medical history will be included. No incentives will be provided for the participants.

At least five focus groups of five patients, each with a duration of 60 min, are planned (a total of 25 patient representatives). The focus groups will differ in terms of age and history of previous therapies. The interviews will be audio-recorded and moderated by experienced interviewers and their assistants.

#### Data analysis

All sessions will be transcribed verbatim and analysed by two investigators, presumably with the qualitative content analysis according to Mayring [10]. The transcribed data will not be linked to any identifying patient information to assure anonymity. We will closely adhere to the Consolidated Criteria for Reporting Qualitative Studies (COREQ) [11]. We will seek ethical approval by the Institutional Review Board of the Ethics Committee of the Medical Faculty of the LMU Munich. Besides, prior to the focus groups, we will collect signed informed consent from each participant.

### Step 3: Survey of practising, board-certified dermatologists

#### Objective

To complete the identified outcomes from the literature search and focus groups, a survey with dermatologists and further training assistants will be conducted to identify practice-relevant endpoints.

#### Design

A questionnaire will be developed de novo based on the results from the literature review in step 1. Dermatologists will be asked to evaluate the need for suitable endpoints in the field of their daily practice. The questionnaire will contain the most frequently reported endpoints from the literature search (step 1), which will be evaluated using a three-tier scale (meaningful, not meaningful, no assessment). Additionally, the dermatologists will have the possibility to name further endpoints in a free-text field that were not reported in the literature search and that will later find their way into the Delphi procedure.

#### Sampling, recruitment, data collection

Dermatologists will be invited electronically through a newsletter of the German Dermatological Society (Deutsche Dermatologische Gesellschaft, DDG) and the Professional Association of German Dermatologists (Berufsverband der Deutschen Dermatologen, BVDD) to participate in the survey. Besides, we will ask potential participants to forward the electronic questionnaire to others whom they regard as having the required expertise (snowball sampling). Overall, there were 5944 active dermatologists in Germany in 2017 [12]. Hence, the estimated sample size for this descriptive study is *n* = 361 with an alpha-error of 5%, a 95% confidence interval and an estimated population of 5944 dermatologists in Germany.

Refusals will not be documented and no incentives will be provided to participants. Each physician is allowed to participate only once. Completed questionnaires will be sequentially numbered for data-entry purposes but will not be linked to any identifying patient information to assure irreversible anonymity. The survey tool LimeSurvey will be used for the creation of the electronic questionnaires (https://www.limesurvey.org/de/).

#### Data analysis

We will perform descriptive analysis of the results, including calculation of frequencies, means, and median. The analyses will be conducted with the help of SPSS (IBM SPSS Statistics version 25, IBM Corporation, Armonk, NY, USA).

### Project 4: Two-step Delphi consensus process

#### Objective

Using the outcomes identified in steps 1–3, an online, electronic Delphi method will be used for developing the preliminary list of outcomes in two rounds.

#### Delphi procedure

The Delphi method facilitates a consensus process by using a series of sequential questionnaires to collect data from a panel of experts and patients on the topic under investigation [5]. This method has been used in various developments of core outcome sets [13–16]. The Delphi process is iterative and based on the scoring of a series of structured statements that are revised, fed back to the participants and repeated in multiple rounds, in increasing detail, until a consensus has been reached [5].

#### Recruitment of participants

National experts in the field of AKs will be recruited within the DDG and the BVDD, as well as among the participating experts of the German S3 guideline ‘Actinic keratosis and squamous-cell carcinoma of the skin’. Besides, we will identify further organisations (e.g. professional societies) relevant to stakeholder groups using personal networks and a web-based search. These contacts will be emailed and asked to propose stakeholders or to disseminate our recruitment letter to their members. Other methods for contacting stakeholders include advertisement at medical conferences and through professional societies for clinicians. Furthermore, we will approach patient representatives and patient support groups for the Delphi procedure. Lastly, we will ask clinicians within our personal networks to identify former patients or caregivers that might be interested in an opportunity to participate in the study and invite them to contact us.

We aim at achieving an interdisciplinary panel consisting of 10% patient representatives and 10% methodologists. The remaining panel members (80%) should have expertise in the field of dermatology and/or oncology.

#### Data collection and analysis

A two-stage electronic Delphi procedure will be conducted, followed by a final face-to-face consensus meeting. For the two sequential first rounds, questionnaires will be completed using the web-based system DelphiManager (http://www.comet-initiative.org/delphimanager/). This programme is designed to facilitate the development/generation and management of Delphi surveys. For both rounds, panel members will receive an email with a link to the questionnaire. Responses will be collected, analysed, and redistributed to all participants with their individual response to each item for further comment in the second round. Each participant will also have access to the previous overall rating of the outcomes. Each round will have a response closing date 21 days after the date of distribution of the survey. Responses will be documented and an electronic reminder will be sent to anyone who has not responded by day 14, with a final reminder after 19 days. The number of participants completing each round and attrition across rounds will be documented. Within the Delphi process, ratings of all panel members will be weighed equally. Panel members who did not enter a round will not be invited for the next round.

#### First round

The first round of the Delphi process will contain the outcomes identified in steps 1–3 with their corresponding definitions as well as a short questionnaire on the demographic data on the participants. The outcomes will be grouped under the four AK-relevant domains, i.e. efficacy, safety, cosmesis, and patient satisfaction. To ensure completeness of outcomes, participants in this round will also be invited to add up to two further ‘novel’ outcomes that they consider critical.

The participants will be asked to rate the outcomes on a 9-point Likert scale (GRADE Working Group). Typically, 1–3 means that an outcome is of limited importance, 4–6 means that it is important but not critical, and 7–9 means that it is critical [17]. Consensus on the need for a particular endpoint means that > 70% of the panel rate the endpoint on the scale as 7–9 and less than 15% as 1–3. Consensus on the dispensability of an outcome means that > 70% of the panel rate the endpoint on the scale as 1–3 and less than 15% as 7–9 (Table 1) [18]. Endpoints where a consensus was reached on their need (rating 7–8) will be moved directly to the consensus meeting. Items where a consensus on the dispensability was reached in round 1 (rating of 1–3) will be excluded from the next round whereas all items where no consensus was reached will be included in the second round.

**Table 1:** Overview on the consensus classification for the Delphi procedure

Consensus	Description	Definition
Consensus in	Consensus that outcome should be included in the core outcome set	70% or more participants scoring 7–9 *and* < 15% participants scoring 1–3
Consensus out	Consensus that outcome should not be included in the core outcome set	70% or more participants scoring 1–3 *and* < 15% of participants scoring 7–9
No consensus	Uncertainty about importance of outcome so retain for next round	Anything else

#### Second round

The second Delphi round will contain all outcomes from round 1 where no consensus had been achieved. Only participants who have been involved in the first round will be invited to round 2. For each outcome from round 1, the rating results (i.e. the proportion of participants rating each point on the 9-point rating scale) will be presented. Each participant will be able to see their previous evaluation score from round 1. Prior to the second Delphi round, all panel members will have the opportunity to comment on their judgement and provide these comments as anonymous feedback to the rest of the panel in round 2, as these comments may help in reaching a consensus when viewing these items from a different perspective. Participants will be asked to re-rate the importance of each outcome with knowledge of their and other group’s previous ratings on the 9-point Likert scale (as in round 1).

### Project 5: Consensus meeting

A full-day in-person meeting is proposed for the final face-to-face consensus meeting. Only the participants who have been involved in the first two rounds of the Delphi process will be invited to the consensus meeting. Materials will be distributed prior to the meeting. All outcomes rated as ‘consensus out’ in the two previous Delphi rounds will be descriptively presented as a set. The panel will be asked if they support the removal of these outcomes. Next, all outcomes judged ‘consensus in’ and ‘no consensus’ will be presented individually. The panel will be asked, following discussion, to vote whether the outcome should be included in the catalogue. All outcomes that are judged neither ‘consensus in’ nor ‘consensus out’ will be discussed individually and the panel will vote whether the outcome should be included.

### Project 6: Dissemination of the outcome catalogue

A publication of the catalogue of the endpoints as original work in an international journal for dermatology is planned and should serve as primary medium for the dissemination of the results. For the preparation of the manuscript, we will closely adhere to the checklist from Williamson et al. for reporting of the development of a core outcome set [5]. Furthermore, a layman-understandable version will be produced. Dissemination will continue to take place via the distributors of the participating professional societies in order to achieve maximum visibility.

## Discussion

The aim of this multi-step process is to develop a catalogue of most relevant, suitable, and standardised endpoints for the treatment of AKs. A core outcome set for the evaluation of interventions for AKs, both in RCTs and in daily practice, is lacking, making it difficult to compare and follow up on different treatment options. Furthermore, the publication landscape of trials on AKs is highly heterogeneous and head-to-head comparisons are sparse. Thus, it is problematic to summarise the existing evidence in meta-analyses and treatment guidelines. Harmonising the outcomes can help to make the results of trials comparable and find a lowest common denominator which should be reported in trials dealing with interventions for AKs. Ultimately, our initiative should contribute to make better and evidence-based treatment decisions and to improve the care of patients with AKs.

The inclusion of affected patients as well as board-certified dermatologists bears huge potential for the development of the outcome catalogue and ensures that all relevant outcomes are considered. However, the individual projects pose some challenges. Possible hurdles for the literature review include the high number of publications on treatment options for AKs and the inconsistent definitions of an endpoint within different studies. A possible limitation for the realisation of the focus groups is the recruitment of participants. Challenges for the questionnaire-based study for treating dermatologists include a possibly low response rate to the questionnaires. Lastly, the challenge for the two-stage Delphi process and the consensus meeting is the organisational and transparent implementation of the procedure. Hence, conflicts of interest must be publically disclosed in advance. If there is a conflict of interest, an abstention from the consensus is desirable. In addition, a low response rate to the questionnaires in the first two Delphi rounds may be possible.

### Project status

The review of the literature has started; the search queries for the three databases have been established. Currently, two independent investigators are performing the title-abstract screening.

## Supplementary information

**Additional file 1.** SPIRIT 2013 Checklist: recommended items to address in a clinical trial protocol and related documents*.

## Data Availability

The datasets that will be created and analysed during the current study are available from the corresponding author on reasonable request.

## Abbreviations

AK: Actinic keratosis
COMET: Core Outcome Measures for Effectiveness
COREQ: Consolidated Criteria for Reporting Qualitative Studies
cSCC: Cutaneous squamous cell carcinoma
MeSH: Medical Subject Headings
RCT: Randomised controlled trial
